# Prevalence and predictors of tuberculin skin positivity in Hellenic Army recruits

**DOI:** 10.1186/1471-2334-6-102

**Published:** 2006-06-23

**Authors:** Vasilios German, Georgios Giannakos, Petros Kopterides, Matthew E Falagas

**Affiliations:** 1 Medical Service of the Hellenic Army Supply and Transportation Corps Training Center, Sparta, Greece; 2 Internal Medicine and Infectious Diseases Department, 417 NIMTS Hospital, Athens, Greece; 3 Alfa Institute of Biomedical Sciences (AIBS), Athens, Greece; 4 Department of Medicine, Tufts University School of Medicine, Boston, Massachusetts, USA

## Abstract

**Background:**

Tuberculosis (TB) remains one of the leading causes of death among infectious diseases worldwide. Despite its low incidence rates in countries of Western Europe and North America, the resurgence of TB in Eastern Europe and the increased immigration from high-incidence countries imply that extreme vigilance is required in order to detect early, treat, and isolate all new cases. In this study, we analyzed the prevalence and predictors of tuberculin skin testing positivity in Hellenic Army recruits.

**Methods:**

The study population consisted of 953 Greek military recruits enlisted inthe Army during the period from November 2005 toFebruary 2006. Tuberculin skin testing was performed on all study subjects upon enrollment, according to the routine procedures. A tuberculin skin test reaction size >15 mm was considered positive for all study participants. Epidemiological data regarding age, repatriation status, geographic area of residence, smoking habits, and parental occupation were collected by means of personal interviews. In addition, body weight, height, and body mass index were measured.

**Results:**

The mean age of the studied subjects (± SD) was 23.5 (± 6.4) years. The overall prevalence of tuberculin positivity was 3.9% (37/953), and bivariable analysis showed that it was associated with lower weight (p = 0.047) and repatriation status (p < 0.001). Tuberculin skin testing was positive in 2.6% of natives (24/900) and 24.5% of repatriates (13/53). A backward, stepwise multivariable logistic regression model showed that only repatriation status was independently associated with tuberculin positivity (p < 0.001; odds ratio [OR]: 14.1; 95% confidence interval [CI]: 6.5–30.3).

**Conclusion:**

While the incidence of tuberculosis in the native Greek population is low, and comparable to other Western European countries, the extremely high tuberculin positivity in repatriated persons underscores the importance of actively screening for TB in order to promptly identify, isolate, and treat cases of active and latent infection.

## Background

Currently, tuberculosis (TB) is one of the leading causes of mortality among infectious diseases worldwide, and the number of new cases continues to rise despite intense eradication efforts. Recent epidemiologic analyses show that the incidence of new TB cases has increased considerably during the last 2 decades in Africa as well as Eastern Europe, while it has decreased in countries of Western Europe as well as USA, Canada, Australia, and New Zealand [1].

We sought to evaluate the prevalence and predictors of tuberculin skin testing positivity in young recruits joining the Hellenic Army the period from November 2005 to February 2006.

## Methods

### Study population

The study took place in Sparta, Greece, where the Supply and Transportation Corps Training Centre of the Hellenic Army is currently based. Military service is obligatory for young men in Greece. Upon enlistment, recruits are asked to undergo a chest x-ray, a routine physical examination and a tuberculin skin testing (TST). The purpose of this program is to prevent recruits with active, highly contagious TB or old, healed TB at risk for progression from enlisting and spreading the disease to other individuals. Our study population consisted of 953 men, aged 17–34 years. The study protocol was approved by the Medical Directorate of the Hellenic Army General Staff.

### Tuberculin skin testing

Two tuberculin units (TU) of purified protein derivative in 0.1 ml (RT 23 in Tween 80, SSI, Statens Serum Institute, Denmark) was injected intradermally on the volar side of the forearm. The reaction was read 48 to 72 hours later and interpreted based on CDC guidelines [2]. Study participants with reaction size greater than 15 mm were there-after interviewed, examined by the staff of the Department of Pulmonary Medicine of 401 Army General Hospital of Athens, Greece, and received treatment for latent tuberculosis infection if appropriate.

### Data collection

Epidemiological data regarding age, repatriation status, geographic area of residence, smoking habits, and parental occupation were collected by means of personal interviews in standardized data collection forms. In addition, body weight, height, and body mass index were measured.

For the purposes of our analysis the residential areas were divided in urban (including seven major cities of Greece: Athens, Thessaloniki, Patra, Iraklion, Larisa, Ioannina and Alexandroupolis), semi-urban (other cities or towns with population >10,000 people), and rural (including all other smaller cities, towns and villages <10,000 people and rural). In addition, repatriates were considered to be those persons of Greek heritage who repatriated to Greece from the former Soviet Republics and Balkan countries.

The socioeconomic level of the recruits' parents was determined according to the Erikson Goldthorpe Portocarero (EGP) social class scheme [3], which classifies occupations in seven classes (with class I including higher grade professionals, class II lower grade professionals administrators, class III routine non-manual employees, class IV small proprietors, artisans, farmers and smallholders, class V lower grade technicians, class VI manual workers, and class VII including unskilled manual workers and agricultural workers). We chose to consider the parental socioeconomic level as representative of the recruits' financial status since, at this young age, most of the recruits were either unemployed or dependent on their parents' financial support. Finally, as no previous medical records were available for review, the recruits' vaccination history remained unknown.

### Statistical analysis

Chi-square test was used in the bivariable analysis for the comparison of the prevalence of tuberculin reactivity in the various subgroups. Normality was assessed and t-test or a non-parametric test was used for normally and non-normally distributed continuous variables. A *p*-value < 0.05 was considered statistically significant. A backward, stepwise multivariable logistic regression model was applied in order to assess relationships between recruits' epidemiological factors and tuberculin reactivity. Variables that were found to be statistically associated with TST positivity in the bivariable analysis (*p*-value < 0.05) were included in this model, based on our study design. Data were analyzed using SPSS for Windows version 12.0 (SPSS Inc, Chicago, IL, USA).

## Results

All recruits were male, and their mean age (± SD) was 23.5 (± 6.4) years. The overall prevalence of tuberculin skin testing positivity was 3.9% (37 of the 953 recruits). In Table 1, we present the results of the bivariable analysis we performed in order to investigate possible associations of the epidemiological characteristics with the presence of TST reactivity. Lower weight (p = 0.047) and repatriation status (p < 0.001) were statistically associated with presence of TST positivity. Of note, TST was found to be positive in 2.6% of natives (24/900) and 24.5% of repatriates (13/53).

**Table 1:** Bivariable analysis of risk factors associated with tuberculin skin testing positivity.

	**TST (-) [N = 916]**	**TST (+) [N = 37]**	**P – value**
**Variables**	**Mean ± SD or n (%)**		

Age, (years)	23.5 ± 6.5	24.3 ± 4.6	0.30
Body weight, (kg)	80.9 ± 14.7	76.3 ± 13.1	0.047
Height, (m)	1.79 ± 0.07	1.78 ± 0.07	0.07
Body-mass index, (kg/m^2^)	25.2 ± 4.4	24.0 ± 4.0	0.10
Smoking, (packets-per-year)	2.8 ± 4.1	3.2 ± 5.4	0.69
Repatriation	40/916 (4.3 %)	13/37 (24.5 %)	<0.001
Current residence			0.07
Urban	445/916 (48.6%)	25/37 (67.6%)	
Semi-urban	203/916 (22.2%)	6/37 (16.2%)	
Rural	268/916 (29.2%)	6/37 (16.2%)	
EGP social class*			0.78
I	28/660 (4.2%)	1/28 (3.6%)	
II	42/660 (6.3%)	3/28 (10.7%)	
III	224/660 (33.9%)	9/28 (32.2%)	
IV	115/660 (17.4%)	3/28 (10.7%)	
V	12/660 (18.2%)	0/28 (0%)	
VI	119/660 (18.0%)	4/28 (14.3%)	
VII	120/660 (18.2%)	8/28 (28.6%)	

Abbreviations: EGP: Erikson Goldthorpe Portocarero social class scheme (it refers to the socioeconomic status of the recruits' parents); TST: tuberculin skin test; SD: standard deviation

* Data were not available for all studied participants

A backward, stepwise multivariable logistic regression model showed that repatriation status was the only parameter independently associated with tuberculin reactivity (p < 0.001, odds ratio [OR]: 14.1; 95% confidence interval [CI]: 6.5–30.3). Residence in a rural part of Greece (p = 0.29) and lower weight (p = 0.19) were not found to be independent predictors of the presence of TST reactivity in the multivariable analysis.

## Discussion

The main finding of our study is that, compared with previous available epidemiologic data from Greece, there has been a significant decrease in the prevalence of TST positivity in Hellenic Army recruits over the last 15 years. Bouros *et al*. previously reported a decline of TST positivity from 14.2% in 1981 to 6.8% in 1991. This seemed to follow the downward trend initially observed in smaller studies conducted during the period from 1934 to 1980 [4-6]. Herein, we report a further decline of TST positivity, which reflects the progress towards tuberculosis elimination made over the past decades in Greece. Furthermore, the prevalence of TST positivity in our study is similar to that reported in recruits and military personnel in USA [7], Italy [8] and Norway [9].

Another interesting finding of our study is the fact that the prevalence of positive TST reactions was almost 10-fold higher in repatriates than non-repatriates recruits. Greece experienced a dramatic population movement towards the former USSR during the civil war that followed the World War II (1946–1949). Soon after the collapse of the former Eastern Block, thousands of persons gradually repatriated. It seems that these subjects, together with immigrants and refugees from countries of Asia and Africa, represent a major reservoir of tuberculosis in Greece, often resistant to multiple anti-tuberculous drugs [10].

The identification of persons with positive TST may serve as an indirect estimate for the overall disease burden, since these patients – after exclusion of those vaccinated against TB – represent a potential "reservoir" of *Mycobacterium tuberculosis*. Previous reports that modeled the relevant epidemiological trends anticipated a further decrease of tuberculin reactivity in Hellenic Army recruits compared with the decades following World War II, based on the initial success of the anti-tuberculosis campaign [4]. However, immigration from developing countries with a high prevalence of TB and massive repatriation from previous Soviet Republics and Balkan countries have challenged the declining trend of TB incidence in Greece, with the additional worrisome feature of multidrug resistant TB cases [10,11].

The Hellenic Army has participated actively in the national campaign against TB for the last 70 years. The goals of disease control and prevention are achieved through diagnosis and treatment of active as well as latent infection. Serial studies on the TST positivity in Army recruits can serve as an indicator of the secular epidemiological trends of mycobacterial burden in the population in time. Our findings support the notion that Greece has taken important steps towards the reduction of TB disease burden. However, it seems that the "Achilles' heel" of national efforts to combat TB might be the lack of expanded screening of immigrants and repatriating persons.

Our study admittedly has a number of limitations. We were unable to obtain any data on prior BCG vaccination. Even though the effect of prior BCG vaccination on future TST positivity is unclear, a positive TST reaction could represent latent TB infection, previous BCG vaccination, or a cross reaction to non-tuberculous mycobacteria. In fact, guidelines from the American Thoracic Society (ATS) advice against taking prior BCG vaccination into account when interpreting a TST reaction [2]. However, the cut-off of the tuberculin skin testing recommended by ATS is more applicable for individual patient management than for an epidemiological study. Furthermore, it is unclear to what extent the higher infection prevalence observed among repatriates in our study reflects a higher proportion of BCG cross-reactions or differences in immunization practices between Greece and other countries. In fact, universal BCG vaccination of all children at school entry or in later school years- meaning at the age of 5 to 7- continues to be the official advice of the public health authorities in Greece.

We chose to use the cut-off of 15 mm according to well-accepted guidelines [2], because all studied recruits had normal chest x-ray, reported no recent close contact with a person with active TB, and were generally in a good state of health; in fact, persons with risk factors for tuberculosis, such as diabetes mellitus, HIV, hematologic malignancies, and immunodeficiency are not usually recruited in the Greek Army. A valid argument can be made against the use of the same cut-off level of 15 mm of induration for defining a positive tuberculin reaction in both non-repatriates and repatriates, considering the recommendation that the cut-off should be 10 mm for recent immigrants from high TB prevalence countries [2], and the fact that the repatriates in our study can generally fit in this group. In accordance with a recently published meta-analysis showing that positive skin tests with indurations of >15 mm are more likely to be the result of tuberculous infection than of BCG vaccination [12], we opted for a cut-off that would provide relatively high specificity for latent TB infection and reduce misclassification bias in any comparative study. Reducing the cut-off to 10 mm for repatriates and keeping it to 15 mm for non-repatriates would introduce substantial bias and further magnify the observed difference in the prevalence of tuberculin skin positivity.

It should be emphasized that our data cannot be easily extrapolated to other populations (e.g. women), as we focused our study in a highly selected group of young, and presumably healthy, males. Nevertheless, we believe that the study population is a representative sample of the general male population of that age in Greece, since enlistment in the Hellenic Armed Forces is obligatory for all males above the age of 18 and all repatriated men under the age of 35. Moreover, the tested population came from all parts of Greece (urban, semi-urban, and rural) and from all social strata. The designation of the training facility for each recruit is random and there is no seasonal variation since the date of enlistment depends only on the birth date. Previous studies in Greece that posed similar research questions with our study included populations with similar characteristics with our studied subjects. This is mainly a result of the fact that the policy for army recruitment has not changed in Greece in the last decades. However, the proportions of Army recruits with positive tuberculin skin testing are not directly comparable between studies conducted in different time periods, mainly because of differences in the interpretation of the test results, including the selected cut-off.

Finally, the tuberculin test is an imperfect test as it is highly "operator-dependent", requires two visits and skilled personnel for placement and interpretation, and does not reliably separate latent TB infection from prior immunization with BCG or infection with environmental mycobacteria. Nevertheless, it remains the standard test of choice for the diagnosis of latent TB infection until we gain more experience with the new tests that quantify interferon-gamma production in response to *M. tuberculosis *antigens [13,14].

## Conclusion

Our findings show the further decline in the prevalence of TST positivity in young Hellenic Army recruits compared to results of previous studies in this population. However, it should be acknowledged that the observed decline in the prevalence of TST positivity may be at least in part due to a change in the selected cut-off used for the interpretation of the test results. In addition, our findings stress the importance of initial screening for latent and active TB infection in people originating from high-incidence countries. The goal of TB elimination could be eventually achieved only if vigilant control measures are actively implemented, especially focusing on persons repatriating, immigrating or seeking asylum from countries of the developing world.

## Competing interests

The author(s) declare that they have no competing interests.

## Funding

None.

## Authors' contributions

VG, PK, and MEF had the idea for the study. VG and GG collected the relevant data. MEF did the statistical analysis. All authors contributed to the writing of the manuscript and approved its final version.

## Pre-publication history

The pre-publication history for this paper can be accessed here:


